# Negative interaction between emotional vulnerability and depressive symptoms may heighten suicidal ideation among Japanese university students: a cross-sectional study

**DOI:** 10.3389/fpsyt.2024.1383907

**Published:** 2024-10-03

**Authors:** Shinji Yamaguchi, Daiki Nagamine, Yuka Murofushi, Kojiro Matsuda

**Affiliations:** ^1^ Division of Public Health, Department of Hygiene and Public Health, School of Medicine, Women’s Medical University, Tokyo, Japan; ^2^ Graduate School of Health and Sports Science, Juntendo University, Chiba, Japan; ^3^ Faculty of Health and Sports Science, Juntendo University, Chiba, Japan; ^4^ Faculty of Management, Josai University, Saitama, Japan

**Keywords:** emotional vulnerability, suicidal ideation, depressive symptoms, mental health, cross-sectional study

## Abstract

**Introduction:**

Studies examining the relationships among suicidal ideation, emotional vulnerability, and depressive symptoms are scarce. This study examined the effects of emotional vulnerability and depressive symptoms on suicidal ideation among Japanese university students

**Methods:**

A questionnaire survey was conducted with 370 Japanese university students. Data were analyzed using descriptive statistics and multiple regression analysis

**Results:**

In the multiple regression analysis, depressive symptoms were significant (*β* = .46, *p* <.001) but emotional vulnerability was not (*β* = .05, *p* = .318). A significant interaction emerged between emotional vulnerability and depressive symptoms (*β* = .22, *p* <.001). The model’s *R* ^2^ value was.31 (*p* <.001). Simple slopes tests revealed the impact of depressive symptoms on suicidal ideation, even with low emotional vulnerability (*b* = .06, *β* = .27, *p* <.001), and a higher impact with high emotional vulnerability (*b* = .15, *β* = .65, *p* <.001)

**Discussion:**

The negative interaction between emotional vulnerability and depressive symptoms may heighten suicidal ideation among Japanese university students. Interventions targeting emotional vulnerability may help reduce suicidal ideation and achieve lower suicide rates.

## 1 Introduction

The period of young adulthood, which includes the college years, is a time when mental health is prone to deterioration owing to factors such as the pursuit of economic independence after higher education and the emergence of new social needs (1, 2). In particular, psychologically vulnerable individuals tend to experience excessive negative emotions and stress, and these tendencies may be associated with symptoms of depression and suicidal ideation. Vulnerability denotes that some people are more affected by stressful life events than are others (3) and may be more vulnerable to psychological problems (e.g., behavioral health problems, neglect-related issues, and emotional impairment).

The phenomenon of being at risk for psychological hurt is referred to as “vulnerability,” and was proposed by Sinclair and Wallston (4) as a psychological construct. They defined it as a “pattern of cognitive beliefs reflecting dependence on achievement or external sources of affirmation for one’s sense of self-worth” (p. 120) (4). In the Japanese context, Hayashi (5) defined vulnerability as the susceptibility to psychological harm and a possible state of fragility or emotional hurt. Vulnerability represents a cognitive belief or experience that renders a person susceptible to hurt feelings in response to everyday life events. Additionally, psychological vulnerability is conceptualized as cognitive patterns—specifically patterns reflecting social dependence, self-oriented perfectionism, negative attributions, and self-blame—that make individuals more susceptible to stress (6). These definitions indicate that vulnerability is a “cognitive belief” (7) about oneself being easily hurt and that it differs from personality traits or states. It is also observed that the more vulnerable people are, the more negative their perceptions of external sources of stress/hurt, thereby indicating how emotional pain can be mediated by negative cognitive styles (8). Additionally, psychological vulnerability can lead to dysfunctional or less functional cognitive, feeling, and behavioral (e.g., passivity, self-blame, isolation, and catastrophizing) patterns, which can in turn lead to psychopathology or decreased psychological well-being (4). Given that vulnerability relates to negative cognitive beliefs, it is considered distinct from positive psychological concepts such as resilience and hardiness. In addition, one study found that maladaptive coping and cognitive emotion regulation strategies, such as behavioral avoidance and dysfunctional attitudes, are vulnerability factors for depression, anxiety, and suicidal tendencies (9).

One theory that incorporates the concept of vulnerability is the hopelessness theory proposed by Beck (10) and Abramson et al. (11). It posits that when individuals with high levels of emotional vulnerability are faced with a negative event, the event is likely to lead to negative outcomes and becomes meaningless to the individual. This theory hence explains how increased levels of emotional vulnerability result in negative reasoning and despair (in relation to the associated events) by individuals, and then lead to depressive symptoms (12). Meanwhile, despair and sadness have been associated with suicidal ideation and increased risk of suicide (13).

Suicide is a serious issue both domestically (in Japan) and internationally. Worldwide, suicide is a major problem among university students, and a higher incidence of suicide is observed among those with mental health problems (14). The same is true in Japan, where the primary cause of death among young people (aged 15–39 years) is suicide (15)—a situation that remains extremely serious to date (16, 17). Specifically, among Japanese people aged 15–39 years, the number of student suicides reached 1,031 in 2021 and increased to 1,063 in 2022 (18). Certain risk factors are known to precede suicide (e.g., suicidal thoughts, low self-esteem, depressive symptoms, and social isolation) (19). Meanwhile, suicidal ideation, also called suicidal thoughts or ideas, refers to various thoughts, desires, and concerns about death or suicide (20). High levels of suicidal ideation are often associated with a greater likelihood of engaging in suicidal behavior. Indeed, Van Heeringen (21) proposed the suicidal process model in which suicide is a successive process that goes from “suicidal ideation” to “suicide attempts,” and then to “completed suicide.” Nevertheless, there is no universally accepted definition of suicidal ideation, which leads to ongoing challenges for clinicians, researchers, and educators (22). Brás et al. (23) described suicide as the result of a suicidal process that typically begins with suicidal ideation and may culminate in a suicide attempt, and that when suicidal ideation is high, there may be an increased risk of suicide attempt or actual suicide.

A relevant theory is the three-step theory proposed by Klonsky and May (24), which explains the conditions under which suicidal ideation and suicide attempts occur. This theory explains the progression from suicidal ideation to suicidal behavior in three steps using four elements (i.e., pain, despair, connectedness, and capability for suicide). In the first step, pain (i.e., psychological, interpersonal, physical, acute, or chronic) and hopelessness are factors that trigger suicidal ideation, meaning that suicidal ideation may tend to decrease when pain is relieved, or when there is an expectation of pain reduction over time or through effort. In the second step, there is the weakening of connectedness, referring to both connections with others and broader connections (e.g., attachment to work, roles, and interests), which then amplifies suicidal ideation. In the third step, there is the capability for suicide; experiencing pain, despair, prolonged distress, and decreased social interactions and connections all result in a greater capability for suicide, which may then lead to suicidal behavior. Corroborating the propositions of the three-step theory, Klonsky et al. (25) reported that emotional misery, psychological pain, and feelings of hopelessness about the future affect suicidal ideation and suicide attempts.

Suicidal ideation, or thinking about suicide without actually making plans to commit suicide (26), has been shown to predict impaired psychosocial functioning, poor future psychological health, and forms of injury-risk behaviors among university students (27). As aforementioned, suicide among university students is a major societal problem, and the associated risk factors include those related to school (e.g., study slump and worries about career paths), health (e.g., depression, schizophrenia, and stress-related disorders), and family problems (e.g., parent–child relationships, discordances, and parental scolding). The number of risk factors outlined here showcases that suicide occurs because of various factors, which may sometimes be interconnected (16). Although it is extremely difficult to predict suicide attempts, which be discussed further in this manuscript, suicidal ideation is one of the factors that can be used to understand suicide risk.

Although individuals may have thoughts of suicide, not all of them may attempt suicide (28), and despite the possibility of using suicidal ideation to predict suicide risk, its isolated use remains an imprecise predictor of suicidal behavior (26). One factor contributing to suicidal ideation is individual vulnerability. However, to date, research has primarily focused on the relationships between only two of the three variables: suicide ideation, depression symptoms, and emotional vulnerability. For instance, studies have examined the associations between emotional vulnerability and depressive symptoms (8, 27) and between depressive symptoms and suicidal ideation (29), but no studies have investigated the association between emotional vulnerability and suicidal ideation. Furthermore, to the best of our knowledge, no studies have simultaneously examined the relationships among emotional vulnerability, depressive symptoms, and suicidal ideation, or the combined influence of the first two on suicidal ideation. One reason for this may be the lack of measures for assessing everyday levels of emotional vulnerability. However, Yamaguchi et al. (8) recently developed the first index for measuring everyday levels of emotional vulnerability. Furthermore, research on emotional vulnerability was initiated in 2022, showcasing the significance of investigating whether experiencing everyday emotional vulnerability and depressive symptoms may influence people’s suicidal ideation levels.

Therefore, this study examined the combined effects of emotional vulnerability and depressive symptoms on suicidal ideation among Japanese university students. Clarifying the relationships among these three variables can contribute to a better understanding of the thought processes that may cause suicidal ideation among Japanese university students experiencing emotional vulnerability.

## 2 Methods

### 2.1 Survey period and participants

A survey was conducted during the first semester of 2022 (i.e., from May to July) at multiple universities in Japan, targeting 370 students (173 of the participants were women, mean age 19.6 years, *SD* = 1.07). The survey was conducted by the university to which the primary author and co-authors are affiliated, and the data were collected from various academic disciplines, including humanities, social sciences, and natural sciences, without bias toward a specific department.

### 2.2 Survey procedures and ethical considerations

The survey was conducted using an online Google Forms questionnaire. The survey’s URL and explanatory text were posted on the online classroom portal of the class taught by the primary author and co-authors. After the class, an announcement about the survey was made. Participation in the survey was voluntary, and it was emphasized that, owing to the anonymous nature of the questionnaire, respondent identities would not be disclosed, and participation would have no impact on academic performance. Participants spent approximately 10 minutes completing the questionnaire.

Students participated in the survey voluntarily, and the first page of the Google Forms questionnaire clearly outlined the survey’s purpose and provided instructions. Participants were required to click the “I understand” button, indicating their written consent after reading the content and deciding to participate in the survey. The first page instructions highlighted the option for participants to discontinue their participation or cooperation in the survey if they felt distressed during the questionnaire, especially in instances involving sensitive inquiries, such as those associated with suicidal thoughts. Additionally, the first page of the survey informed participants that they could seek counseling from a psychiatrist or psychologist if they felt down or distressed after completing the survey. Because all the survey items were marked as mandatory, there were no unanswered questions or omissions, as participants could not proceed to the next page if responses were not provided on the previous one.

This study was conducted after obtaining approval from the research ethics committee of the university with which the primary author is affiliated (Approval No: 2021-108). Furthermore, this study adhered to the ethical standards outlined in the 1964 Declaration of Helsinki and its subsequent amendments.

### 2.3 Measurements

#### 2.3.1 Sociodemographic data

Regarding demographic data, the questionnaire included items on gender, age, grade, and department of enrollment.

#### 2.3.2 Emotional vulnerability

We used the 16-item Emotional Vulnerability Scale developed by Yamaguchi et al. (8) to assess everyday emotional vulnerability. This scale includes the following four factors, each with four items: vulnerability toward criticism or denial, vulnerability toward worsening relationships, vulnerability toward interpersonal discord, and vulnerability toward procrastination and emotional avoidance. Example items are “I get hurt when my opinion is criticized” and “I feel hurt when I put off things I do not like.” Items were rated on a four-point Likert scale ranging from 1 (I completely disagree) to 4 (I completely agree). Scores were calculated by averaging the scores for the items, whereby the higher the score, the higher the level of emotional vulnerability. According to the original study, Cronbach’s α of the scale ranged from.79–.85 (8). The range was.77–.88 in this study, indicating that the scale was as reliable as in the original study. According to Yamaguchi et al. (8), the construct validity of the scale was acceptable: GFI = .94, AGFI = .91, CFI = .96, and RMSEA = .05.

#### 2.3.3 Depressive symptoms

The 20-item Self-Rating Depression Scale developed by Zung (30) was used to assess depressive symptoms. This single-factor scale includes items including “I feel downhearted and blue,” “I have crying spells or feel like it,” and “I get tired for no reason.” Items were rated on a four-point scale, ranging from 1 (A little of the time) to 4 (Most of the time), where the higher the score, the higher the level of depressive symptoms. Cronbach’s α of the scale was.73 in the original study (20) and.84 in this study, demonstrating its reliability. The total score was calculated as the sum of all item scores.

#### 2.3.4 Suicidal ideation

We used the six-item, single-factor Short-Form Suicidal Ideation Scale developed by Sueki (31) to assess suicidal ideation. Items such as “Do you wish to die?” and “How often do you have suicidal thoughts?” are included in the scale. The descriptions in the response scales differed across items, but all response scales ranged from 0–2 points. Scores were calculated by totaling the scores of all items, whereby the higher the score, the higher the level of suicidal ideation. This scale, developed in Japan, is available in Japanese and allows for an evaluation with a minimal number of items. Cronbach’s α of the scale was.89 in the original study (31) and.81 in this study, thereby demonstrating its reliability. Moreover, the area under the curve was.78, and extant findings revealed that scale scores have predictive validity for suicidal ideation (32). The total score was calculated as the sum of all item scores.

### 2.4 Data analysis

Descriptive statistics were calculated for participants’ sociodemographic data and all other main study variables. We subsequently conducted a multiple regression analysis to examine how vulnerability, depressive symptoms, and their combination influence suicidal ideation. Considering that gender differences in vulnerability and depressive symptoms have been identified in previous studies (8, 27, 30), emotional vulnerability, depressive symptoms, and the interaction term (emotional vulnerability × depressive symptoms) were set as independent variables, with gender as a covariate and suicidal ideation as the dependent variable. The analysis was performed using a forced-entry method. The interaction term was created by standardizing the scores for emotional vulnerability and depressive symptoms (mean = 0, standard deviation = 1) and obtaining the z -score by multiplying both variables (33). When the interaction term was significant, a simple slopes analysis was conducted. The significance level was set at 5%, and the effect size was assessed using *R* ^2^. All analyses were conducted using SPSS version 28.0.

## 3 Results

First, we calculated demographic data and descriptive statistics for each variable. Participants’ sociodemographic data are summarized in **Table 1**. The means and standard deviations were 2.70 (± 0.61) for emotional vulnerability, 40.3 (± 8.98) for depressive symptoms, and 1.22 (± 2.10) for suicidal ideation.

**Table 1:** Participants’ sociodemographic data.

Variable	N	(%)
Gender		
Male	197	53.2
Female	173	46.8
Age (in years)		
18	21	5.7
19	204	55.1
20	76	20.5
21	42	11.4
22	19	5.1
23	8	2.2
Year of study		
First year	115	31.1
Second year	170	45.9
Third year	31	8.4
Fourth year	54	14.6
Departments		
Humanities	87	23.5
Natural Sciences	249	67.3
Social Sciences	34	9.2

In the results of the multiple regression analysis (**Table 2**), only depressive symptoms (*β* = .46, *B* = .11, *p* <.001, *t* = 9.37, 95% CI of *β* [.083 -.128]) were significant, whereas emotional vulnerability (*β* = .05, *B* = .17, *p* = .318, *t* = 1.00, 95% CI of *β* [-.165 -.507]) did not reach statistical significance. Additionally, there was a significant interaction between emotional vulnerability and depressive symptoms (*β* = .22, *B* = .40, *p* <.001, *t* = 4.97, 95% CI of *β* [.241 -.558]). The model’s coefficient of determination was *R* ^2^ = .30 (*p* <.001). Given the significant interaction, simple slopes tests were conducted. Interestingly, even when emotional vulnerability was low, depressive symptoms had a consistent impact on suicidal ideation (*b* = .06, *β* = .27, *p* <.001), and the impact was stronger when emotional vulnerability was high (*b* = .15, *β* = .65, *p* <.001).

**Table 2:** Multiple regression analysis results for the comparison of emotional vulnerability and suicidal ideation.

Variable	Suicidal ideation	
	*β*	95%CI
Emotional vulnerability	.057 ^n.s.^	-0.038–0.152
Depression	.457^***^	0.361–0.553
Emotional vulnerability × Depression	.220^***^	0.133–0.307
*R* ^2^	.309^***^	

***p <.001. CI, Confidence Interval; n.s., not significant.

## 4 Discussion

This study examined the effects of emotional vulnerability and depressive symptoms on suicidal ideation among Japanese university students. Based on the results of the multiple regression analysis, suicidal ideation was not significantly associated with emotional vulnerability, but it was significantly associated with depressive symptoms and the interaction between emotional vulnerability and depressive symptoms. This indicates that depressive symptoms influence suicidal ideation. Moreover, the significant interaction term suggested that suicidal ideation increases not only with depressive symptoms but also through the synergistic effect of emotional vulnerability and depressive symptoms. In previous studies, the impact of depressive symptoms on suicidal ideation has been consistently documented (34–36).

The novelty of this study lies in its indication that the interaction between emotional vulnerability and depressive symptoms influences suicidal ideation. The results of simple slopes analyses revealed significance at the 0.01% level for emotional vulnerability at both *±* 1 *SD*, suggesting that suicidal ideation scores tend to be higher in cases of severe depressive symptoms regardless of emotional vulnerability. For example, individuals with higher emotional vulnerability exhibited increased suicidal ideation levels associated with the presence of depressive symptoms compared to those with lower emotional vulnerability. This suggests that experiencing emotional vulnerability in response to negative emotional experiences or events intensifies any feelings of sadness and depression, consequently strengthening the desire to die.

Vulnerability represents a negative cognitive tendency that is negatively associated with positive affect, life satisfaction, and optimism (4). Vulnerability, depressive symptoms, and suicidal ideation—the negative psychological concepts explored in this study—show positive associations, as demonstrated in both this and previous studies. Conversely, optimism, self-esteem, and resilience are positive psychological concepts, and research shows that self-esteem (23, 37) and resilience (38) function as protective factors against suicide risk. In hopelessness theory, individuals prone to emotional hurt from events tend to engage in negative inferencing, leading to feelings of hopelessness and depressive symptom onset. Therefore, the sequence “vulnerability → hopelessness → depressive symptoms” appears reasonable according to this theory. Although the current study did not address hopelessness, our findings suggest that the interaction between emotional vulnerability and depressive symptoms is associated with suicidal ideation. This is corroborated by hopelessness theory, wherein emotional vulnerability is associated with feelings of hopelessness and depressive symptoms and which posits that severe depressive symptoms and mood stagnation may lead to suicidal ideation.

As aforementioned, we observed a significant effect of the interaction term of emotional vulnerability and depressive symptoms, a finding that contradicts the non-significance of the main effect of vulnerability in the multiple regression analysis. These results collectively imply that vulnerability alone is not directly associated with suicidal ideation and that even when hurt by an event, individuals may not necessarily entertain thoughts of wanting to die. This is possibly because such thoughts, despite occurring momentarily, are likely to still be perceived as transient. After being hurt, people experience stress responses that may impair mental health, and when these responses become severe, suicidal ideation arises (39). These findings may explain the non-significance of the main effect of vulnerability and the significance of the interaction term and demonstrate that there is the likelihood—but not the certainty—for people to feel like they want to die when they are hurt.

The vulnerability index used in this study was developed in a previous study (8) to measure how vulnerable people are to daily life events. An examination of the contents of the scale items (see the examples in the Methods section) showed that individuals who are more vulnerable to the following factors may tend to perceive events more negatively and be negatively influenced mentally: being criticized for their opinions, experiencing friendship deterioration, and being insulted. These events can leave individuals psychologically hurt and cause emotional pain. Research shows that negative experiences such as victimization from friends and peers, conflict, isolation, and loneliness increase suicidal thoughts (40), implying that these hurt-related situations can give rise to feelings of wanting to die or committing suicide. A study that examined risk and protective factors for suicidal ideation showed a positive impact of negative life events on suicidal ideation (23), supporting this idea. In that study, negative life events included psychological abuse (humiliation), separation, and loss. These events tend not to be recurrent in daily life, and the psychological pain they evoke may be stronger than that caused by victimization by peers or isolation; therefore, we suggest that such events can indeed have an impact on suicidal ideation. Furthermore, a previous study reported a positive correlation between pain and feelings of hopelessness, indicating that these factors influence suicidal ideation (41). Therefore, when individuals experience some form of psychological pain, heightened emotional vulnerability likely exacerbates the impact of this pain, leading to increased feelings of despair and, consequently, elevated suicidal ideation.

Thus far, researchers in Japan have used single-item yes-or-no scales and multiple-item self-developed scales to assess suicidal ideation (42). As such, it was desirable for a new study on the topic to use a scale with reliability and validity, regardless of the number of items in the scale. To address this research gap, we measured suicidal ideation using the short-form Suicidal Ideation Scale developed by Sueki (31). This scale is simple, contains only six items, and has been proven reliable and valid in previous studies, thereby addressing the problem associated with the use of non-reliable and non-validated assessment tools for measuring suicidal ideation levels in Japanese research.

In summary, this study showcases the importance of considering the mental health of university students when tackling suicidal behaviors in this population, and that understanding their level of emotional vulnerability may help combat suicide ideation in Japan. Given the alarming trend of suicide ranking as the leading cause of death among youth in Japan (15), it is imperative to apply insights gleaned from research to implement early interventions and bolster mental health education. Considering the impact of the interaction between susceptibility to distress (as assessed by emotional vulnerability) and depressive symptoms on suicidal ideation, as shown in our study, it may be vital to identify at-risk students and provide timely support. In particular, considering that we assessed vulnerability to daily life events—situations that anyone may recurrently encounter—it is conceivable that individuals more susceptible to these experiences may be more prone to depressive symptoms. If their vulnerability and/or depressive symptoms are severe, this could escalate to suicidal ideation.

These delineations underscore the importance of understanding vulnerability in daily life as a crucial concept in mental health prevention and care for university students. Moreover, enhancing the accuracy of mental health information among both youth and adults is crucial. Strengthening mental health awareness campaigns through educational institutions and media platforms can foster environments where young people feel comfortable discussing mental well-being and seeking assistance. By collectively focusing on the vulnerability individuals experience in their daily lives, we may contribute to addressing issues related to suicidal ideation and prevention.

### 4.1 Limitations and directions for future research

This study has three limitations. First, this was a one-time cross-sectional survey. It is possible that the scores for vulnerability, depressive symptoms, and suicidal ideation were influenced by specific life events and stressful situations that occurred close to or at the time of the survey. Additionally, while demographic data such as gender, age, academic year, and faculty affiliation were collected, medical history and the presence of mental disorders were not assessed. Considering that depression could also be a risk factor for suicide, future research should gather demographic data and consider conducting longitudinal studies to examine fluctuations and impacts over time.

Second, social desirability bias and recall bias might have affected the results of this study. We inquired about sensitive topics vulnerability, depressive symptoms, and suicidal ideation, but there is a tendency for respondents to present themselves in a more favorable light due to social desirability (43). This means that even if an individual feels suicidal, they might convince themselves that they do not feel this way or underestimate their vulnerability, to avoid being perceived negatively. Additionally, since the study was conducted retrospectively using a self-administered questionnaire, recall bias may have introduced errors in the results, as participants might not have accurately remembered or reported past experiences. Therefore, future research should include measures to account for both social desirability and recall bias.

Third, we treated depressive symptoms as a typical stress response. However, subsequent reactions to being hurt may include poor general mental health, feelings of helplessness, anger, and hopelessness. Future research could examine whether similar results can be found when other mental health factors and feelings of hopelessness are considered.

## 5 Conclusions

This study investigated the relationships among emotional vulnerability, depressive symptoms, and suicidal ideation in a sample of Japanese university students. Specifically, this study focused on the ways in which emotional vulnerability and depressive symptoms synergistically influence suicidal ideation. The findings suggested that emotionally vulnerable people are more likely to exhibit depressive symptoms and engage in suicidal ideation. This study underpins the importance of providing mental health care services to university students exposed to emotional vulnerability before their mental health deteriorates further.

## Data Availability

The raw data supporting the conclusions of this article will be made available by the authors, without undue reservation.
